# Taurochenodeoxycholic acid promotes abdominal fat deposition by modulating the crosstalk between bile acid metabolism and intestinal microbiota in broilers

**DOI:** 10.1186/s40104-025-01280-z

**Published:** 2025-10-30

**Authors:** Xi Sun, Chaohui Wang, Xiaoying Liu, Yun Li, Zhouzheng Ren, Xiaojun Yang, Yanli Liu

**Affiliations:** College of Animal Science and Technology, Northwest A&F University, Yangling, 712100 China

**Keywords:** Abdominal fat, Broiler, Cecal microbiota, Taurochenodeoxycholic acid

## Abstract

**Background:**

The role of bile acids in modulating the gut microbiota and their impact on host metabolism has garnered significant attention. Taurochenodeoxycholic acid (TCDCA) is the predominant bile acid within the chicken bile acid pool and is closely related to metabolic disorders. The current study aims to investigate the potential effects of TCDCA on abdominal fat deposition in broilers. From 14 to 28 days of age, the broilers in the CON group received an oral administration of 1 mL of saline, while those in the treatment groups were administered 1 mL of a solution containing 0.05 g, 0.10 g, or 0.20 g of TCDCA.

**Results:**

The results showed that TCDCA treatments from 14 to 28 d had no significant effects on BW, ADFI, ADG and FCR in broilers at the age of 28 days of age. However, the abdominal fat percentage in the 0.20 g TCDCA group significantly increased, accompanied by higher TBA and HDL-c levels, as well as a reduction in apolipoprotein B levels in serum. In addition, serum triglyceride levels tended to be higher in the 0.20 g TCDCA group (*P* = 0.098). The 0.20 g TCDCA treatment increased the gene expressions of *SREBP-1*, *C/EBP-α*, and *ELOVL6*, while decreasing the mRNA abundance of *ATGL* and *CPT-1* in the abdominal fat. Serum levels of TCDCA, TDCA, and THDCA were significantly higher after 0.20 g TCDCA administration, while TCA levels were significantly lower, as determined by the targeted bile acid metabolomics analysis. Conversely, hepatic mRNA levels of *CYP7A1*, *CYP27A1*, *BAAT*, and *BSEP* were increased in the 0.20 g TCDCA group. The oral administration of 0.20 g TCDCA also upregulated the expression of *FXR*, *VDR*, and *FGF19* in abdominal fat. The 16S rRNA analysis of cecal microbiota revealed that a decrease in the Shannon and Simpson indexes in the 0.20 g TCDCA group, and an increase in the Firmicutes/Bacteroidetes ratio. LEfSe analysis revealed that the predominant bacteria in the CON group were *Streptococcus* and *Oscillospira* at the genus level, while *Lactobacillus*, *Parabacteroides*, *Anaeroplasma*, and *Helicobacter* were identified as the dominant genera in the 0.20 g TCDCA group. Functional predictions for the gut microbiota exhibited that lipid metabolism, replication and repair pathway were enhanced in the 0.20 g TCDCA group. Correlation analysis demonstrated that the abundance of *Lactobacillus* was positively correlated with serum levels of TCDCA, THDCA, and TDCA, while the abundance of *Streptococcus* and *Oscillospira* showed a positive correlation with serum TCA levels.

**Conclusion:**

Overall, this study elucidates that the intervention of 0.20 g TCDCA may promote abdominal fat deposition by activating bile acid receptors in abdominal fat, and concurrent alterations in both the intestinal microbial community and bile acid profile.

**Graphical Abstract:**

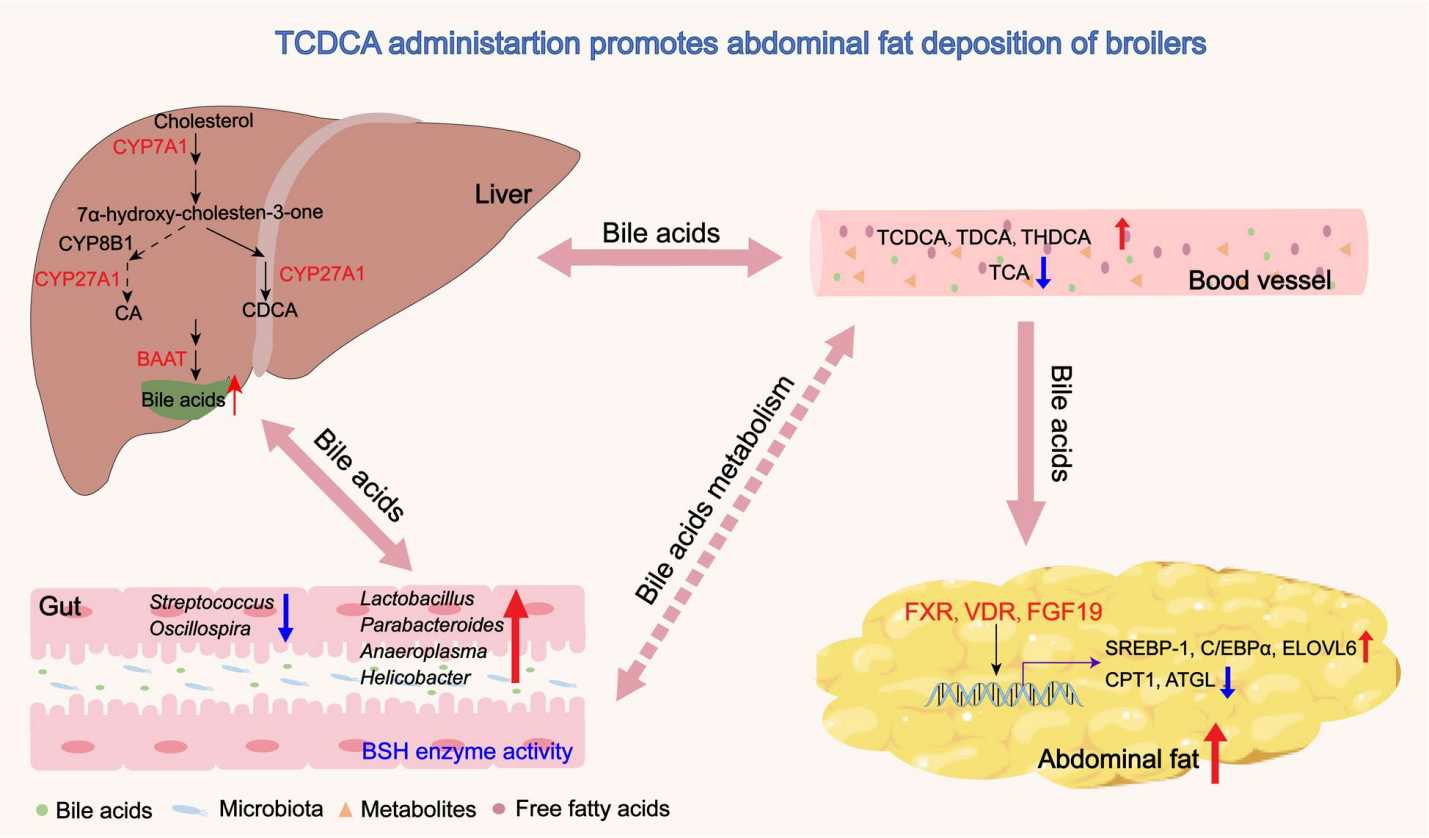

**Supplementary Information:**

The online version contains supplementary material available at 10.1186/s40104-025-01280-z.

## Background

Bile acids are amphiphilic molecules synthesized from cholesterol in the liver that play a crucial role in regulating lipid homeostasis [1]. These molecules can function as signaling agents, modulating lipid metabolism through bile acid receptors present in various tissues [2]. Recent evidence suggests that bile acids can affect adipose tissue deposition [3, 4]. For instance, the inclusion of pig bile powder has been shown to reduce hepatic fat deposition by participating in the microbiota-bile acid profile in broiler chickens [5]. Dietary supplementation with 60 and 90 mg/kg of porcine bile acids has been reported to decrease abdominal fat percentage and enhance serum lipid metabolism as well as cecal microbiota composition in laying hens [6]. Furthermore, hyodeoxycholic acid (HDCA) supplementation has been associated with the promotion of lipolysis and a reduction in backfat thickness in finishing pigs [7]. It is noteworthy that certain bile acids may promote lipid deposition [8]. Lithocholic acid (LCA) serves as an indicator for fatty liver disease [9], and the addition of LCA has been observed to increase white adipose tissue weight in mice without affecting overall body weight [8]. Importantly, there exists a diverse array of bile acids among different animals, and their regulatory effects on lipid metabolism are not consistent [10]. Therefore, it is essential to investigate the effects of individual bile acids on lipid metabolism.

Bile acids play a pivotal role in the digestion and absorption of lipids within the intestine [11]; however, their functions extend beyond lipid metabolism to influence gut microbiota composition and maintain intestinal integrity [2, 12]. Secondary bile acids are recognized as metabolites of microbiota and are involved in a complex interaction with the gut microbiota [13]. Recently, the role of bile acids in modulating the gut microbiota and their impact on host metabolism has garnered significant attention [14]. The modified bile acids possess the capacity to regulate the composition of the microbiota, thereby affecting host metabolism [15]. This exemplifies the communication between gut microbiota and the host, establishing a direct link between microbial activity, fat digestion, and metabolic regulation [16, 17]. The interplay between bile acids, gut microbiota, and lipid metabolism represents a compelling area of research with implications for understanding lipid disorders in broilers. Nevertheless, substantial knowledge gaps persist regarding the specific microbiota taxa involved in bile acid biotransformation [18].

In general, the composition of bile acids exhibits significant variation among different species. For instance, mice predominantly produce muricholic acid, while pigs are known to produce HDCA [19]. More interestingly, a substantial proportion of HDCA (76%) may contribute to the inherent resistance of pigs to type 2 diabetes [3]. Disorders in lipid metabolism and metabolic crosstalk concerning blood glucose levels elevate the risk of type 2 diabetes by diminishing insulin sensitivity [20, 21]. In contrast, chickens demonstrate an exceptionally high proportion of taurochenodeoxycholic acid (TCDCA; 85%) [22]. Although bile acids are typically regarded as beneficial components for lipid metabolism, emerging evidence indicates that TCDCA may be linked to metabolic disorders [23–25]. Consequently, we hypothesized that the high levels of TCDCA in poultry may predispose them to metabolic diseases or lipid metabolism disorders [26]. Therefore, the present study aims to investigate the effects of exogenous TCDCA supplementation on lipid metabolism in broilers, with a particular focus on the perspectives of microbiota and bile acid metabolism.

## Methods

### Animal management and sample collection

The animal experiment conducted in the present study was reviewed by the Animal Care and Use Committee of the College of Animal Science and Technology at Northwest A&F University (Shaanxi, China) and was performed in accordance with the “Guidelines for Experimental Animals” established by the Ministry of Science and Technology, Beijing, P. R. China (Permit number: DK2024023). A total of 144 one-day-old Arbor Acres broilers were used in this study and were randomly assigned to 4 groups, each consisting of 9 replicates, with each replicate containing 4 chickens. From d 1 to 14, the total body weight and feed intake of broilers per cage were recorded to calculate growth performance, including body weight, average daily feed intake, average daily gain, and feed conversion ratio. From the age of 14 to 28 d, the broilers in the CON group received an oral administration of 1 mL of saline daily using pipettes, while the birds in the other three groups were administered 1 mL of a solution containing 0.05 g, 0.10 g, or 0.20 g of TCDCA, respectively. The detailed composition of the basal diet is outlined in Table S1. Following the oral administration treatment, one chicken was randomly selected from each of the replicate pens on d 28 of the trial. Prior to the euthanasia of the broilers, blood samples were collected from the brachial vein, after which the birds were euthanized via cervical dislocation and subsequently dissected. The abdominal fat was excised and weighed, with the abdominal fat percentage expressed relative to body weight (g/g). Fresh cecal chyme was collected for subsequent analysis of gut microbiota. After sampling, the abdominal fat, liver, and cecal content samples were rapidly frozen in liquid nitrogen and subsequently stored at −80 °C for further analysis.

### Serum biochemical analysis

The collected blood samples were subjected to centrifugation at 3,000 × *g* for 10 min to isolate the serum. Biochemical analyses were performed using an automatic biochemical analyzer (BIOBASE, BK-400, Shandong). The measured parameters included total bile acid (TBA), apolipoprotein A1 (APOA1), apolipoprotein B (APOB), triglycerides (TG), total cholesterol (TC), high-density lipoprotein cholesterol (HDL-c), and low-density lipoprotein cholesterol (LDL-c).

### Serum bile acid metabolomics

A targeted metabolomics profiling of bile acids in serum samples was performed using an ultra-performance liquid chromatography coupled with tandem mass spectrometry (UPLC-MS/MS) system (ACQUITY UPLC-Xevo TQ-S, Waters Corp., USA). The targeted metabolomics analysis was conducted by Personal Biotechnology Co., Ltd. (Shanghai, China). In brief, serum samples were vortexed with an ice-cold methanol containing internal standards to obtain the supernatant. Following the addition of a 50% pre-cooled methanol solution, the samples were centrifuged at 4,000 × *g*, and the supernatant from each sample was combined with the internal standard and sealed for subsequent UPLC-MS/MS analysis. Instrument parameters were set in accordance with a previous study [27]. Initially, the derivative samples underwent quality control. Subsequently, raw data were generated by UPLC-MS/MS and further processed using QuanMET software v2.0 (Metabolo-Profile, Shanghai, China) for peak integration, calibration, and quantification of each identified metabolite. The signal intensities were standardized to an internal standard employing the best-matched internal standard method, and outliers were eliminated through z-score filtering. The metabolomic characteristics were compared against the criteria for the target metabolite, which is classified as a metabolite with an MSI confidence level of 1.

### RT-PCR

Total RNA was extracted from the liver and abdominal fat tissue using the AG RNAex Pro RNA Reagent (AG21101, Agbio, China) in accordance with the manufacturer’s protocol. Following a quality assessment conducted via the BioAnalyzer 2100 (Nanodrop ND-1000, Thermo Fisher Scientific), 500 ng of total RNA was reverse transcribed into cDNA employing the Evo M-MLV RT Master Mix kit (AG11706, Agbio, China), in accordance with the manufacturer’s instructions. The relative expression levels of mRNA were quantified using the SYBR Green Pro Taq HS II kit (AG11741, Agbio, China) on the FQD-96A (Bioer, Hangzhou, China). Melting curve analysis was performed to verify the specificity of the qPCR products. The relative expression levels of target genes were calculated using the 2^−ΔΔCt^ method, with β-actin serving as the internal reference gene. Comprehensive information regarding the primer sequences is provided in Table S2. For a detailed description of the experimental procedures, please refer to the previous article [28].

### 16S rRNA analysis

Detailed methodologies are described in a previous publication [29]. Genomic DNA was extracted from cecal microbiota utilizing a commercial DNA extraction kit (Tiangen, China). The 16S rRNA gene, specifically targeting the hypervariable regions V3–V4 of bacteria, was amplified and purified with Agencourt AMPure Beads (Beckman Coulter, USA). The 16S rRNA sequencing was conducted by Shanghai Personal Biotechnology Co., Ltd. (China) on the Illumina platform (San Diego, USA). The raw data underwent quality filtering and then aligned against the Greengene 13 database. Alpha and beta diversity analyses were conducted and visualized through Quantitative Insights Into Microbial Ecology 2 (QIIME2) [30] and principal coordinate analysis. Differentially abundant taxa were identified using Linear Discriminant Analysis Effect Size (LEfSe) [31], applying a threshold of linear discriminant analysis > 2 and *P* < 0.05. Predictive functional profiling of microbial communities was annotated in the Kyoto Encyclopedia of Genes and Genomes database and visualized using STAMP software.

### Statistical analysis

All data are presented as mean ± SEM. The SEM values were calculated based on the means, using the formula: SEM = SD/√n, where SD denotes the standard deviation. The General Linear Model program of SPSS version 27 (Chicago, IL, USA) was employed to analyze the data through one-way ANOVA, normality distribution tests, and tests for homogeneity of variance (Levene’s test). A *P*-value of less than 0.05 is considered statistically significant. The* P*-values (0.05 < *P* < 0.10) illustrated in the figure indicate a trend between the comparison groups. The bar graphs were generated using GraphPad Prism version 8 (Boston, USA).

## Results

### The effects of TCDCA administration on growth performance and serum biochemical indices in broilers

**Fig. 1:**
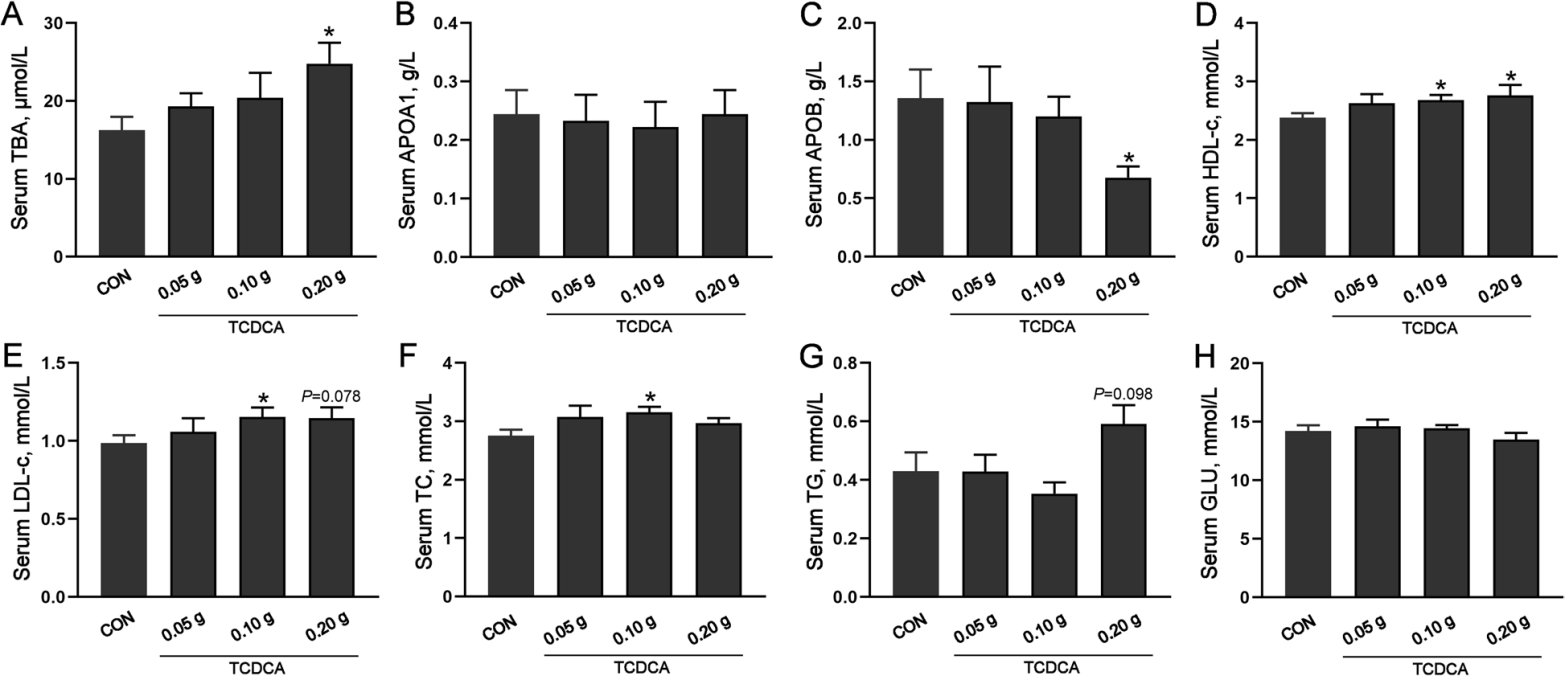
The effects of administration of TCDCA on serum biochemical indicators in broilers. **A**–**H** Serum TBA, APOA1, APOB, HDL-c, LDL-c, TC, TG, and GLU levels. ^*^P < 0.05 and ^**^P < 0.01 are denoted the statistical significance. In the treatment group of broilers aged 14 to 28 d; the CON group was oral administration with normal saline, and the TCDCA treatment group was administrated 0.05 g, 0.10 g, and 0.20 g of TCDCA, respectively (*n* = 9 individuals)

As illustrated in Table 1, there was no difference about growth performance among the four groups. Meanwhile, TCDCA treatments from d 14 to 28 did not affect body weight, average daily feed intake, average daily gain, or feed conversion ratio in broilers. However, the percentage of abdominal fat was significantly higher following the administration of 0.20 g TCDCA on d 28 when compared to the other three groups (*P* < 0.05). Furthermore, in comparison to the CON group, the serum TBA level in the 0.20 g TCDCA group was significantly higher, while the abundance of the lipid transport-related factor APOB was significantly lower (*P* < 0.05; Fig. 1A–C). The serum levels of HDL-C were significantly higher in the 0.10 g and 0.20 g groups, and LDL-c levels were significantly higher in the 0.10 g group (*P* < 0.05; Fig. 1D and E). Indicators reflecting circulating lipid metabolism revealed that TC levels were significantly upregulated in the 0.10 g TCDCA group, and TG levels exhibited a tendency to be higher in the 0.20 g TCDCA group (*P* = 0.098; Fig. 1F and G). No significant differences were observed in other serum biochemical indices across all groups (Fig. 1H). Based on a comprehensive evaluation of abdominal fat percentage and serum total bile acid concentrations, the 0.20 g TCDCA dosage was selected for subsequent analysis.

**Table 1:** Effects of administration of TCDCA on growth performance in broilers^1^

Items		Group				SEM	*P*-value
		Con	0.05 g	0.10 g	0.20 g		
1–14 d	BW, g	557.64	574.56	568.55	569.05	0.004	0.408
	ADFI, g	41.12	42.40	41.44	43.36	0.001	0.435
	ADG, g	16.97	18.18	17.75	17.79	0.000	0.408
	FCR	1.17	1.21	1.10	1.15	0.017	0.137
14–28 d	BW, g	1,626.94	1,593.33	1,587.59	1,576.11	0.015	0.702
	ADFI, g	109.10	104.77	103.09	107.52	0.002	0.543
	ADG, g	76.38	72.77	72.79	71.93	0.001	0.454
	FCR	1.57	1.60	1.64	1.79	0.042	0.254
1–28 d	Abdominal fat, %	0.91^b^	0.89^b^	0.95^b^	1.18^a^	0.038	0.016
	ADFI, g	75.11	72.63	73.75	72.58	0.004	0.996
	ADG, g	46.68	44.17	45.85	42.70	0.003	0.979
	FCR	1.37	1.39	1.36	1.46	0.037	0.766

*BW* Body weight, *ADFI* Average daily feed intake, *ADG* Average daily gain, *FCR* Feed conversion rate

^1^The different letters (a and b) represent significant differences between different groups (*P* < 0.05). The BW, ADFI, ADG, and FCR using pens as the experimental unit (*n* = 9 pens). Only the abdominal fat percentage data is statistically analyzed based on individual units (*n* = 9 individuals)

### The effects of 0.20 g TCDCA administration on the expression of genes associated with lipid metabolism in abdominal fat

**Fig. 2:**
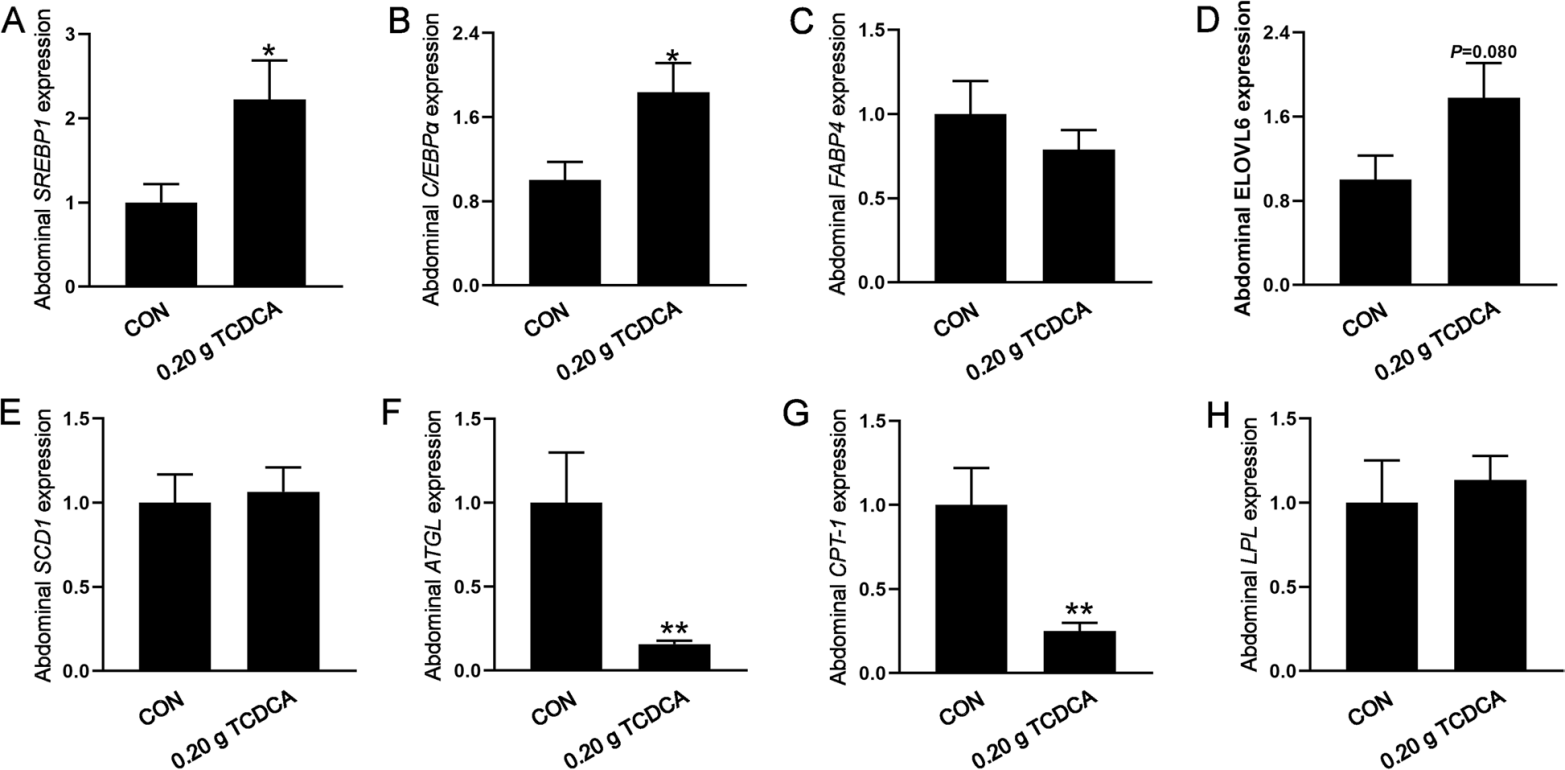
The effects of 0.20 g TCDCA administration on the expression of lipid metabolism-related genes in abdominal fat. **A**–**H** The relative expression of *SREBP1*, *C/EBPα*, *FABP4*, *ELOVL6*, *SCD1, ATGL*, *CPT-1*, *LPL*. ^*^P < 0.05 and ^**^P < 0.01 are denoted the statistical significance. In the treatment group of broilers aged 14 to 28 d; the CON group was oral administration with normal saline, and the TCDCA treatment group was administrated 0.20 g of TCDCA (*n* = 9 individuals)

Considering that phenotypic results suggested a potential role of 0.20 g TCDCA in promoting lipid deposition, we further examined the expression of genes involved in lipid metabolism in abdominal adipose tissue. Genes that are positively correlated with adipocyte differentiation and lipid accumulation, including sterol regulatory element-binding protein 1 (*SREBP-1*) and CCAAT enhancer-binding protein alpha (*C/EBPα*), were significantly upregulated in the abdominal fat of the 0.20 g TCDCA group (*P* < 0.05). Additionally, expression of elongase of very long chain fatty acids 6 (*ELOVL6*) tended to be higher in the 0.20 g TCDCA group (*P* = 0.080; Fig. 2A–E). In contrast, genes implicated in lipolysis, such as adipose triglyceride lipase (*ATGL*) and carnitine palmitoyltransferase I (*CPT1*), exhibited a significant reduction in the 0.20 g TCDCA group (*P* < 0.05; Fig. 2F–H). However, other indicators, including fatty acid binding protein 4 (*FABP4*), stearoyl-CoA desaturase-1 (*SCD1*), and lipoprotein lipase (*LPL*), did not exhibit significant differences between the two groups.

### The effects of 0.20 g TCDCA administration on the composition of serum bile acids

**Fig. 3:**
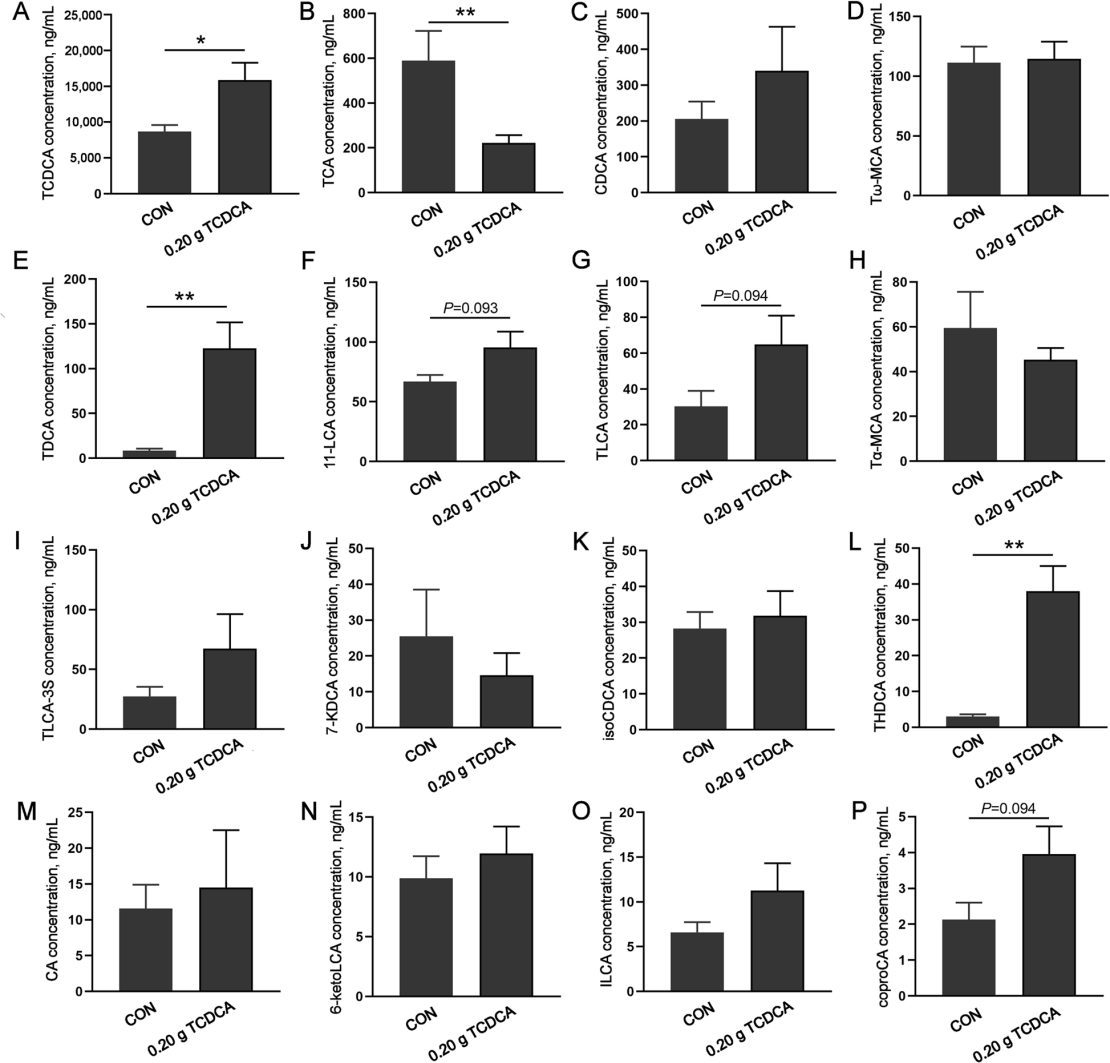
The effects of 0.20 g TCDCA administration on serum bile acids composition. **A**–**T** The serum bile acids including TCDCA, TCA, CDCA, Tω-MCA, TDCA, 11-LCA, TL-CA, Tα-MCA, TLCA-3S, 7-KDCA, isoCDCA, THDCA, CA, 6-ketoLCA, ILCA, and coproCA, are ranked according to their content from high to low. ^*^P < 0.05 and ^**^P < 0.01 are denoted the statistical significance. In the treatment group of broilers aged 14 to 28 d; the CON group was oral administration with normal saline, and the TCDCA treatment group was administrated 0.20 g of TCDCA (*n* = 9 individuals)

To investigate changes in the bile acid pool following the administration of 0.20 g TCDCA, serum bile acid composition was compared between the CON and TCDCA groups (Fig. 3). Compared to the CON group, the concentrations of TCDCA, taurodeoxycholic acid (TDCA), and taurohyodeoxycholic acid (THDCA) were significantly higher in the 0.20 g TCDCA group (*P* < 0.05). Conversely, the level of taurocholic acid (TCA) was significantly lower (*P* < 0.05). Furthermore, various forms of LCA (11-LCA, Tauro-LCA) and copro-cholic acid concentrations, exhibited a tendency to be higher in the 0.20 g TCDCA group (0.05 < *P* < 0.10), while no significant differences were observed in the composition of other bile acids.

### The effects of 0.20 g TCDCA administration on bile acid metabolism and bile acid receptors in the liver and abdominal fat

**Fig. 4:**
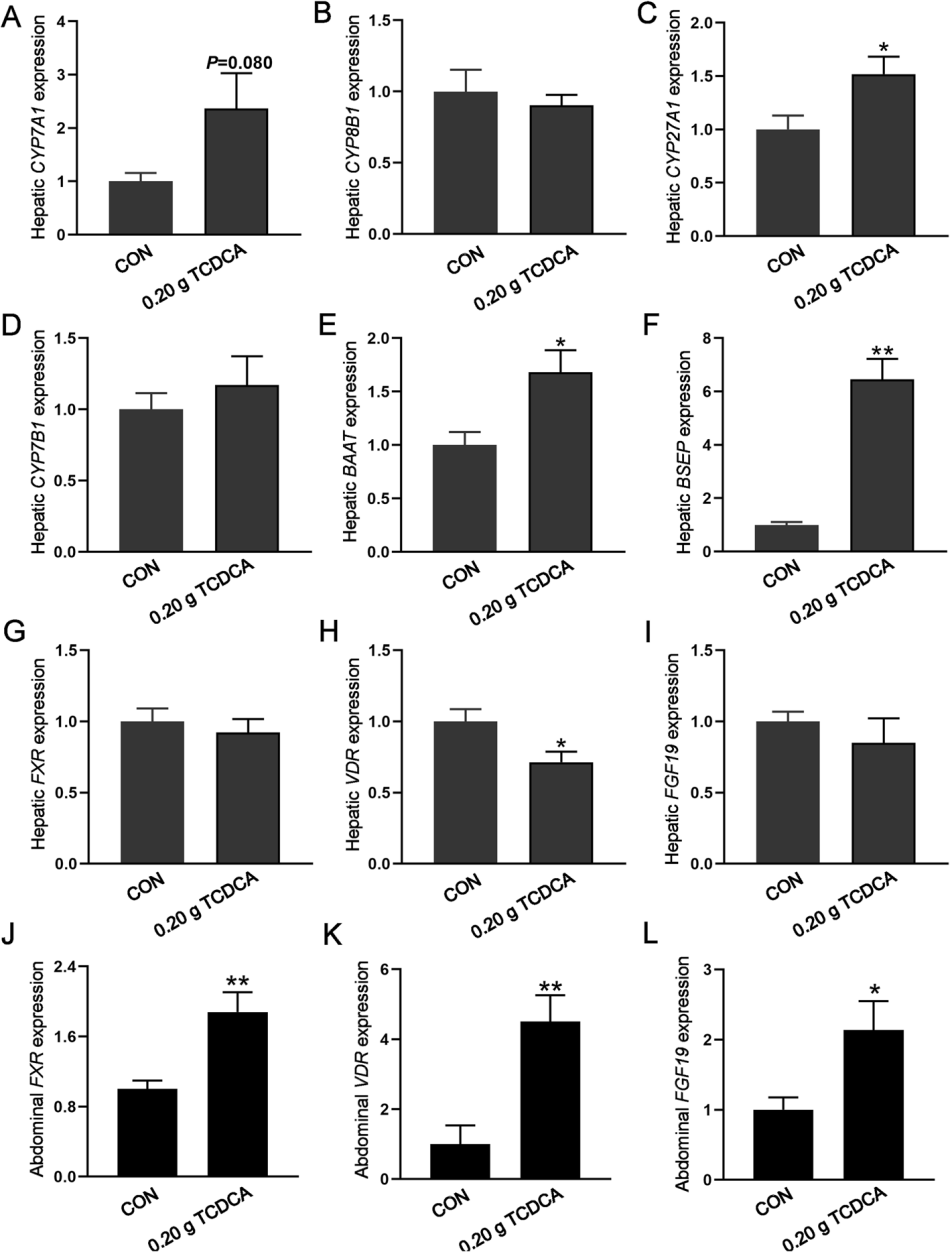
The effects of 0.20 g TCDCA administration on bile acid metabolism and bile acid receptor in the liver and abdominal fat. **A**–**F** The relative expression of genes related to bile acid metabolism in the liver, including *CYP7A1*, *CYP8B1*, *CYP27A1*, *CYP7B1*, *BAAT *and *BSEP*. **G**–**L** The relative expression of genes related to bile acid receptor in the liver (**G**–**I**), and abdominal fat (**J**–**L**), including *FXR*, *VDR*, and *FGF19*. ^*^P < 0.05 and ^**^P < 0.01 are denoted the statistical significance. In the treatment group of broilers aged 14 to 28 d; the CON group was oral administration with normal saline, and the TCDCA treatment group was administrated 0.20 g of TCDCA (*n* = 9 individuals)

To investigate the effects of TCDCA on bile acid metabolism, we analyzed the expression of genes involved in bile acid synthesis, transport, and bile acid receptors. As illustrated in Fig. 4A–D, the hepatic expression of key bile acid synthesis enzymes, specifically cytochrome P450 7A1 (*CYP7A1*) tended to be higher in the 0.20 g TCDCA group (*P* = 0.080), and cytochrome P450 27A1 (*CYP27A1*) was significantly upregulated in the 0.20 g TCDCA group (*P* < 0.05). In contrast, cytochrome P450 8B1 (*CYP8B1*) and cytochrome P450 7B1 (*CYP7B1*) did not demonstrate any significant differences between the CON and 0.20 g TCDCA groups. Furthermore, genes involved in bile acid conjugation and secretion, including bile acid-CoA:amino acid N-acyltransferase (*BAAT*) and bile salt export pump (*BSEP*), were significantly upregulated in the 0.20 g TCDCA group (*P* < 0.05; Fig. 4E and F). Regarding bile acid receptors (Fig. 4G–I), the expression of the vitamin D receptor (*VDR*) in the liver of the TCDCA group was significantly downregulated (*P* < 0.05). Moreover, the bile acid receptors farnesoid X receptor (*FXR*), and *VDR*, along with their downstream effector fibroblast growth factor 19 (*FGF19*), was significantly upregulated in the abdominal adipose tissue of the 0.20 g TCDCA group (*P* < 0.05; Fig. 4J–L). No significant differences were detected in other indices.

### 16S rRNA analysis of differential microbiota and functional predictions in the cecum of broilers

**Fig. 5:**
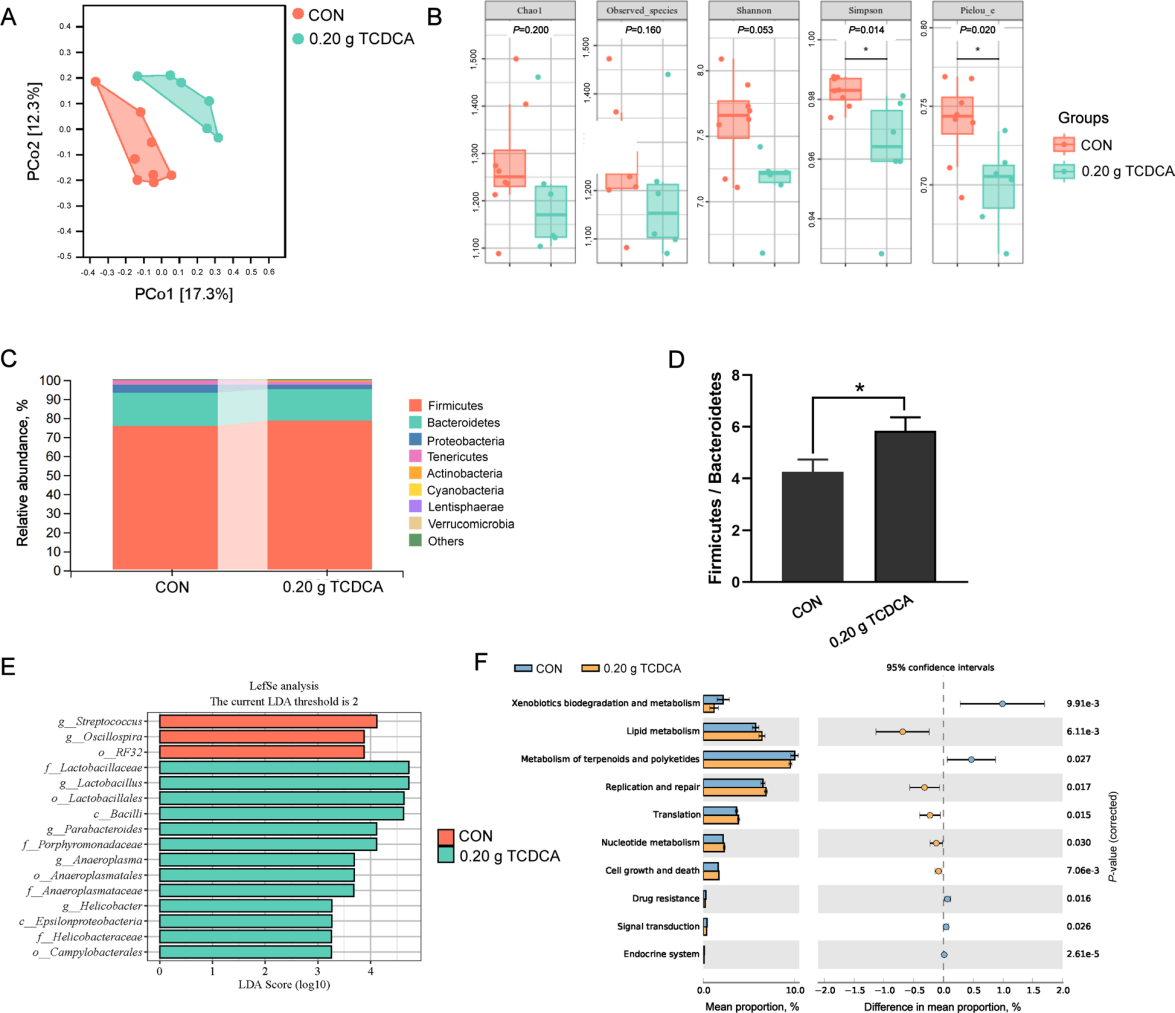
16S rRNA analysis of differential microbiota and functional prediction in the cecum of broilers after 0.20 g TCDCA administration. **A** Principal coordinate analysis of cecal microbiota. **B** Analysis of microbial α-diversity including Simpson index, Pielou's Evenness index, Shannon index, Chao 1 index and Observed_species index. **C** Differences of cecal flora at phylum level. **D** The bar chart of Firmicutes/Bacteroides ratio. **E** LEfSe analyzed the differences in microbial abundance between the CON and TCDCA groups. **F** The functional predictive analysis of the cecal microbiota at level 2. ^*^P < 0.05 and ^**^P < 0.01 are denoted the statistical significance. In the treatment group of broilers aged 14 to 28 d; the CON group was oral administration with normal saline, and the TCDCA treatment group was administrated 0.20 g of TCDCA (*n* = 9 individuals)

To enhance our understanding of the interactions between the host and gut microbiota, as well as the potential role of bile acids, we further investigated the alterations in the cecal microbiota composition. A total of 1,535,525 raw reads were generated, resulting in 1,129,116 clean reads after quality control and merging. On average, each sample yielded approximately 80,651 clean reads. The range of sequence depths across samples spanned from 68,139 to 92,701X (Additional file 1). The results revealed that principal coordinate analysis showed significant differences and distinct separations in microbial communities between the CON and 0.20 g TCDCA groups (Fig. 5A). Analysis of α-diversity demonstrated that the Simpson index, which reflects species diversity, and the Pielou's Evenness index, which reflects evenness, were significantly lower in the 0.20 g TCDCA group (*P* < 0.05). Additionally, the Shannon index exhibited a tendency to be lower in the 0.20 g TCDCA group (0.05 < *P* < 0.1). However, no significant differences were observed between the two groups regarding the Chao 1 index and the Observed_species index (Fig. 5B). At the phylum level, the abundance of Firmicutes, Bacteroidetes, and Proteobacteria ranked as the top 3, with a significant increase in the Firmicutes/Bacteroidetes ratio observed in the 0.20 g TCDCA group (*P* < 0.05; Fig. 5C and D). In the CON group, the dominant genera were *Streptococcus* and *Oscillospira*, whereas *Lactobacillus*, *Parabacteroides*, *Anaeroplasma*, and *Helicobacter* were predominant in the 0.20 g TCDCA group (Fig. 5E). Functional predictions of cecal microbiota at level 2 in the 0.20 g TCDCA group showed enhanced lipid metabolism, nucleotide metabolism, cell growth and death, as well as replication and repair, when compared to the CON group (Fig. 5F).

### The effects of 0.20 g TCDCA administration on the strains encoded by bile salt hydrolase, as well as the correlation analysis between cecal bacteria and serum bile acids

**Fig. 6:**
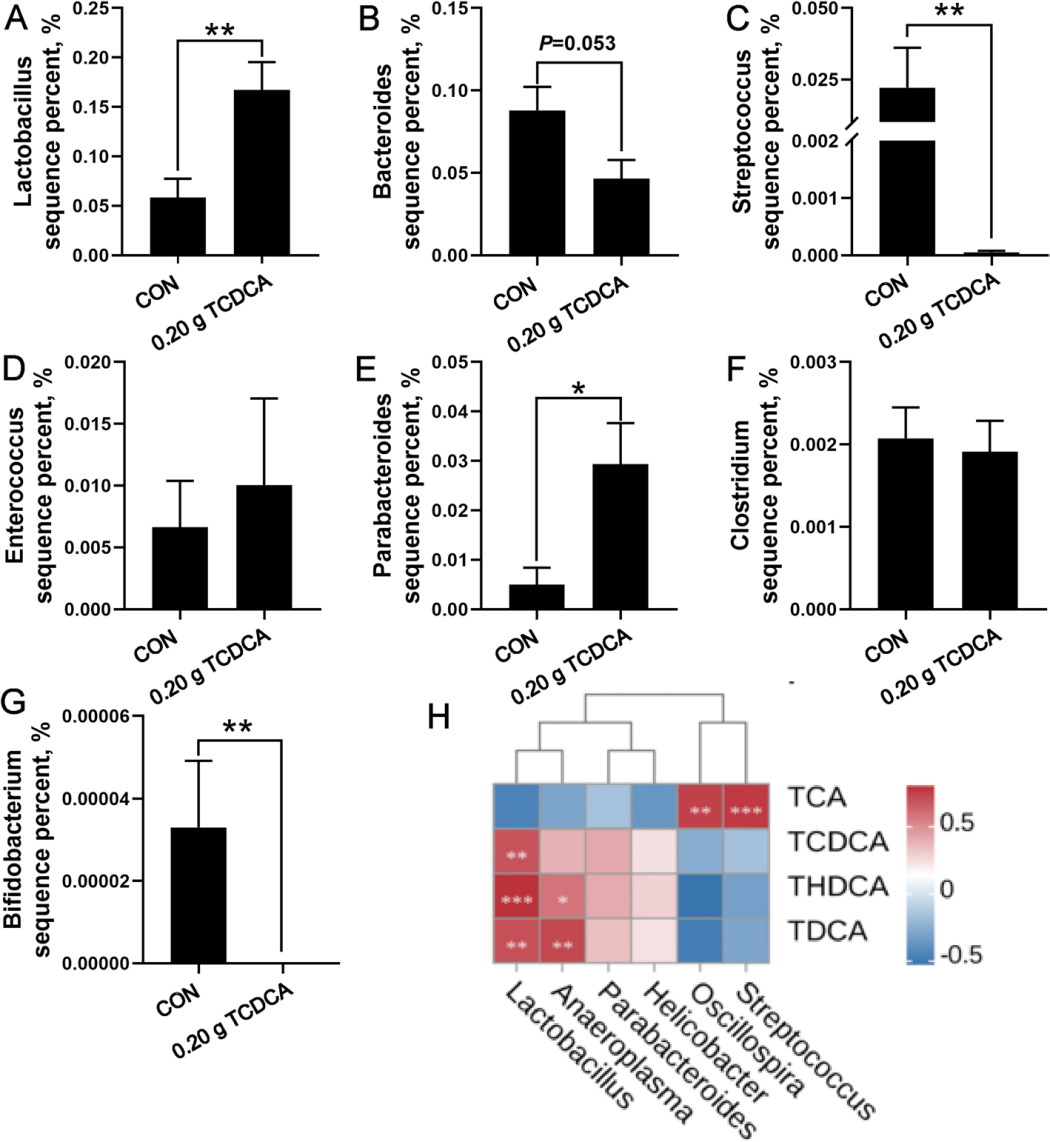
The effects of 0.20 g TCDCA administration on the strains encoded by BSH enzymes and the correlation analysis between cecal bacteria and serum bile acids. **A**–**G** The relative abundance of bacterial genera, including *Lactobacillus*, *Bacteroides*, *Streptococcus*, *Enterococcus*, *Parabacteroides*, *Clostridium*, and *Bifidobacterium*, which have the ability to encode BSH. **H** The correlation analysis between intestinal differential bacteria and serum bile acids. ^*^P < 0.05 and ^**^P < 0.01 are denoted the statistical significance. In the treatment group of broilers aged 14 to 28 d; the CON group was oral administration with normal saline, and the TCDCA treatment group was administrated 0.20 g of TCDCA (*n* = 9 individuals)

The analysis of microbiota encoding bile acid hydrolases in the cecum showed that the administration of 0.20 g TCDCA significantly reduced the abundance of *Streptococcus* and *Bifidobacterium*, and *Bacteroides* tended to be lower in the 0.20 g TCDCA group. Additionally, the abundance of *Lactobacillus* and *Parabacteroides* was significantly enhanced in the cecum of broilers (*P* < 0.05; Fig. 6A–G). Correlation analyses were subsequently conducted to elucidate the relationship between cecal microbiota and serum bile acids in broilers (Fig. 6H). A significant positive correlation was observed between the abundance of *Lactobacillus* in the cecum and the levels of TCDCA, THDCA, and TDCA in the serum. Furthermore, the abundance of *Anaeroplasma* was positively correlated with THDCA and TDCA, as well as a significant positive correlation was also identified between serum levels of TCA and the abundance of *Streptococcus* and *Oscillospira* in the cecum.

## Discussion

Emerging evidence indicates that bile acids play a crucial role in regulating lipid transport [32] by emulsifying and combining with lipids to form chylomicrons [33], which facilitate the absorption of fats and cholesterol [34]. In this study, the administration of 0.20 g of TCDCA daily led to an increased percentage of abdominal fat at D28, accompanied by elevated levels of TBA in the serum. Since elevated levels of TBA can enhance lipid uptake [35], suggesting that the increased percentage of abdominal fat may contribute to the role of bile acids in emulsifying lipids within the intestine. Moreover, evidence has demonstrated that the alterations in bile acid pool hydrophobicity can modulate the transcriptional expression of key genes involved in lipid metabolism [36]. Serum APOB levels are generally positively correlated with the content of TG [37]. In this study, both APOB and TG contents showed an increase, and this phenomenon was also observed in patients with insulin resistance [38]. HDL-c promotes the reverse cholesterol transport, while LDL-c mediates the peripheral transport of cholesterol [39]. Here, both HDL-c and LDL-c in the serum were elevated, which potentially due to reduced clearance of HDL-c, which is to some extent consistent with the phenotype of increased bile acid synthesis. Furthermore, genes positively correlated with lipogenesis, such as *SREBP-1*, *C/EBPα*, and *ELOVL6*, exhibited upregulation in abdominal fat in this study, while the lipolysis-related genes, including *ATGL* and *CPT-1*, were suppressed by the administration of 0.20 g TCDCA. These findings suggest that bile acid metabolism interacts closely with lipid metabolism, with 0.20 g TCDCA supplementation promoting abdominal fat deposition in broilers.

Previous studies have reported that conjugated bile acid species, specifically taurine-conjugated bile acids, are elevated in individuals diagnosed with type 2 diabetes [40]. The concentrations of microbiota-derived secondary bile acids exhibit significant differences between obese and lean pigs, which may play a role in lipid accumulation in obese pigs [41]. In the present study, the bile acid profile in the 0.20 g TCDCA supplementation group was characterized by higher concentrations of specific bile acids, including TCDCA, TDCA, and THDCA, whereas a lower concentration of TCA was observed. As for bile acid absorption, oral administration of 500 mg chenodeoxycholic acid has been shown to enhance bile acid absorption and excretion [42]. Notably, taurine-conjugated bile acids exhibited comparable absorption to unconjugated bile acids [43]. Consistent with this, serum TCDCA levels nearly doubled following oral administration in our study, confirming efficient absorption. Among these differential abundant bile acids, TDCA has been reported to correlate with the insulin clearance rate [44], and its abundance is positively associated with fasting blood glucose and insulin levels. Serum concentrations of THDCA are elevated in chickens exhibiting fatty liver [45], and THDCA has the potential to enhance the secretion of TCDCA, thereby mitigating the hepatotoxicity induced by TCDCA [46]. Additionally, TCA can activate Takeda G protein-coupled receptor 5 (TGR5) in adipose tissue, resulting in suppressed thermogenesis [47]. Furthermore, studies indicate that TCDCA, TCA, TDCA, and THDCA can be interconverted through bile salt hydrolase (BSH) and hydroxysteroid dehydrogenases [48]. Previous studies have consistently demonstrated that bile acid supplementation significantly alters the composition of the host’s bile acid pool [30]. These variations in bile acid composition suggest that TCDCA supplementation may influence the enterohepatic circulation and lipid metabolism, thereby contributing to an increase in abdominal fat percentage; however, the specific mechanisms underlying this phenomenon remain to be elucidated.

To analyze the response of the hepatic and adipose tissue to the administration of TCDCA, we examined the gene expression involved in bile acid metabolism and its receptors. Following the intervention of 0.20 g TCDCA, the enzymes responsible for bile acid synthesis, conjugation, and secretion in the liver, including *CYP7A1*, *CYP27A1*, *BAAT*, and *BSEP*, exhibited upregulation. A similar phenomenon was also observed in the context of bile acid overload, where CYP7A1 continued to increase, which may reflect an adaptive response to elevated bile acid levels [49]. The FXR functions as a principal regulator of bile acid homeostasis and hepatic-intestinal circulation [50]. Certain bile acids possess the ability to activate FXR, thereby modulating the expression of bile acid synthase [51], and inhibiting gluconeogenesis and glycolysis [47]. In various organs, the activation of VDR may exert tissue-specific effects. Research has demonstrated that VDR inhibits the expression of BSEP [52], and the impairment of the VDR specifically in hepatocytes results in increased lipid storage in the liver and a reduction in fatty acid oxidation [53]. In the present study, it was consistently observed that the hepatic VDR levels were inhibited following a 0.20 g TCDCA intervention, while the expression of *BSEP* was upregulated. More importantly, VDR has been shown to influence adipogenesis in adipose tissue [54], and the activation of VDR in adipose tissue is associated with insulin resistance and the expression of lipolysis-related genes [55]. Furthermore, C/EBPα may bind to the gene loci of the VDR to regulate its expression [56]. Consistent with these findings, the current study observed elevated expression levels of both *C/EBPα* and *VDR* in abdominal fat, suggesting that TCDCA may promote the abdominal fat deposition through the activation of the *C/EBPα*-*VDR*. Additionally, FGF19, activated by FXR, can mediate dietary lipid absorption [57], and the white adipose tissue in FGF19 transgenic mice is reduced [58], indicating that the activation of FGF19 exerts a lipid-lowering effect. Similarly, in this study, the *FXR*-*FGF19* axis was activated in abdominal fat following the intervention of 0.20 g TCDCA. These findings reveal that 0.20 g TCDCA promotes abdominal fat deposition by activating bile acid receptors (*VDR* and *FXR*) in adipose tissue, thereby enhancing adipogenic differentiation while suppressing lipolysis.

Extensive research has established a bidirectional relationship between bile acids and gut microbiota [59], with studies demonstrating that bile acid supplementation can significantly alter microbial community structure [60]. The higher activity of BSH reduces bile salt reabsorption, thereby diminishing cholesterol metabolism and nutrient absorption efficiency [61]. Elucidating the interplay between gut microbiota, BSH activity, and broiler chicken physiology may yield critical insights for improving poultry production strategies. In this study, α-diversity indicated that the Simpson index, Pielou's Evenness index, and Shannon index exhibited a decrease, suggesting a reduction in species diversity and evenness following TCDCA administration. Variations in gut microbiota composition significantly affect host health and productivity [62]. The Firmicutes/Bacteroidetes ratio, a marker associated with obesity [63], increased in the 0.20 g TCDCA group, consistent with the observed increase in abdominal fat deposition. Bile acids selectively inhibit or promote the proliferation of certain microbiota [64]. *Streptococcus* exhibits BSH activity, which can mitigate the disturbances caused by a high-fat diet [65]. *Oscillospira* is acknowledged as one of the primary producers of short-chain fatty acids [66], which exhibit a negative correlation with liver fat deposition [67]. A strong positive correlation was observed between serum TCA levels and the abundance of both *Streptococcus* and *Oscillospira*, while the abundance of these genera, which negatively correlates with lipid metabolism, was found to be lower in the TCDCA group. This may indicate internal factors contributing to the elevated abdominal fat rate in the 0.20 g TCDCA group. Additionally, existing studies indicate that the BSH level in the ileum of piglets is predominantly influenced by the abundance of *Lactobacillus* [68]. In this study, the abundance of *Lactobacillus* was positively correlated with serum concentrations of TCDCA, THDCA, and TDCA. These findings suggest that the high abundance of the BSH genus (*Lactobacillus*) in the TCDCA group contributes to the elevated levels of secondary bile acids in the serum. *Parabacteroides* may also enhance BSH activity, inhibit intestinal FXR signaling, and reduce TCDCA levels [69]*,* suggesting that *Parabacteroides* could function as a compensatory mechanism to decrease TCDCA abundance. Notably, the levels of *Anaeroplasma* are positively associated with both obesity and insulin resistance [70], was more abundant in the 0.20 g TCDCA group, which aligns with the observed increase in abdominal fat deposition. Moreover, our data demonstrates a positive correlation between the abundance of *Anaeroplasma* and the levels of THDCA and TDCA. BSH activity critically supports intestinal microbiota adaptation by mediating the resistance of commensal bacteria to bile acids [71]. These findings suggest that specific bacterial taxa may actively engage in the biotransformation of bile acids within the gut, and the administration of TCDCA may create a more favorable environment for the growth and metabolism of *Lactobacillus*, *Parabacteroides,* and *Anaeroplasma*, while concurrently suppressing the proliferation of *Streptococcus* and *Oscillospira*. Notably, specific bacterial taxa were selectively enriched or depleted following TCDCA supplementation, highlighting the pivotal role of bile acid-microbiota interactions in mediating the effects of bile acid supplementation.

Given that the majority of the differential genera enriched in the LEfSe analysis demonstrated BSH enzyme activity, we further characterized the abundance of BSH-synthesizing bacteria. In this study, the administration of 0.20 g TCDCA resulted in a reduction in the abundance of *Streptococcus, Bifidobacterium*, and *Bacteroides*, while the abundance of *Lactobacillus* and *Parabacteroides* increased. The functional prediction analysis revealed that pathways related to lipid metabolism, cell growth and death, nucleotide metabolism, as well as replication and repair, were significantly modulated by TCDCA supplementation. These findings suggest that substantial concentrations of TCDCA may enhance fat emulsification, suppress microbiota abundance [72], and induce DNA damage [73], thereby facilitating more energy flow to the host [74]. The observed enhancement of lipid metabolic pathways in the KEGG analysis further corroborates TCDCA’s role in the regulation of lipid metabolism.

## Conclusion

In summary, this study elucidates that TCDCA modulates lipid metabolism in broilers by activating the bile acid receptors in abdominal adipose tissue, and concurrent alterations in both the intestinal microbial community (particularly BSH-active genera) and the bile acid profile. These findings support the notion that microbiota-bile acid crosstalk plays a significant role in promoting abdominal fat deposition, which may elucidate the susceptibility to lipid metabolism disorders in chickens. More importantly, numerous studies have reported the beneficial effects of bile acids in alleviating metabolic lipid disorders (particularly hyodeoxycholic acid). However, it is crucial to recognize that just like vitamins, different vitamin (A, D, E, K or B) exhibit their own distinct biological functions; different bile acids also possess unique and specialized roles, emphasizing the importance of distinguishing between individual bile acid and bile acid mixture to avoid misunderstanding or overstate bile acid effects. In addition, future research should focus on identifying small molecules antagonist of TCDCA, as well as elucidating light on how TCDCA interacts with gut microbes and affect lipid metabolism.

## Supplementary Information

Additional file 1. The detailed information regarding the 16S rRNA sequencing data processing.

Additional file 2: Table S1. Composition and nutrient levels of experimental diets. Table S2. Forward and reverse primer sequences for PCR analysis.

## Data Availability

The composition and nutritional levels of the experimental diets, as well as the forward and reverse primer sequences for PCR analysis, are presented in Supplementary Material. Additional information for the 16S rRNA sequencing is shown in Additional file 1.

## Abbreviations

APOA1: Apolipoprotein A1
APOB: Apolipoprotein B
ATGL: Adipose triglyceride lipase
BAAT: Bile acid-CoA:amino acid N-acyltransferase
BSEP: Bile salt export pump
BSH: Bile salt hydrolase
C/EBPα: CCAAT enhancer-binding protein alpha
CPT1: Carnitine palmitoyltransferase I
CYP27A1: Cytochrome P450 27A1
CYP7A1: Cytochrome P450 7A1
CYP7B1: Cytochrome P450 7B1
CYP8B1: Cytochrome P450 8B1
ELOVL6: Elongase of very long chain fatty acids 6
FABP4: Fatty acid binding protein 4
FGF19: Fibroblast growth factor 19
FXR: Farnesoid X receptor
HDCA: Hyodeoxycholic acid
HDL-c: High-density lipoprotein cholesterol
LCA: Lithocholic acid
LDL-c: Low-density lipoprotein cholesterol
LEfSe: Linear discriminant analysis effect size
LPL: Lipoprotein lipase
QIIME2: Quantitative insights into microbial ecology 2
SCD1: Stearoyl-CoA desaturase-1
SREBP-1: Sterol regulatory element-binding protein 1
TBA: Total bile acid
TC: Total cholesterol
TCA: Taurocholic acid
TCDCA: Taurochenodeoxycholic acid
TDCA: Taurodeoxycholic acid
TG: Triglycerides
THDCA: Taurohyodeoxycholic acid
TGR5: Takeda G protein-coupled receptor 5
UPLC-MS/MS: Ultra-performance liquid chromatography coupled with tandem mass spectrometry
VDR: Vitamin D receptor
