# Supplementary material for: Taurochenodeoxycholic acid promotes abdominal fat deposition by modulating the crosstalk between bile acid metabolism and intestinal microbiota in broilers
Source: J Anim Sci Biotechnol. 2025 Oct 30;16:143. doi: 10.1186/s40104-025-01280-z (PMC12574242; doi:10.1186/s40104-025-01280-z)
Supplement: Supplementary file 2 — Additional file 2: Table S1. Composition and nutrient levels of experimental diets. Table S2. Forward and reverse primer sequences for PCR analysis. [file 40104_2025_1280_MOESM2_ESM.docx]

**Supplemental Table**

Table S1 Composition and nutrient levels of experimental diets

| Items | Values |
| --- | --- |
| Ingredients (%) |  |
| Corn | 62.00 |
| Soybean meal | 24.50 |
| Corn powder | 4.45 |
| Corn bran | 4.00 |
| Limestone | 1.35 |
| Dicalcium phosphate | 1.23 |
| Premix^1^ | 1.00 |
| L-Lysine sulphate (70%) | 0.58 |
| Vegetable oil | 0.30 |
| Sodium chloride | 0.30 |
| Choline chloride (50%) | 0.10 |
| Preservatives | 0.10 |
| L-threonine | 0.06 |
| DL-Methionine | 0.03 |
| Total | 100.00 |
| Nutrient levels |  |
| Metabolism energy (kcal/kg) | 2810 |
| Crude protein (%) | 18.51 |
| Ca (%) | 1.00 |
| Total P (%) | 0.59 |
| Digestible P (%) | 0.35 |
| Lysine (%) | 1.20 |
| Methionine (%) | 0.43 |
| Methione + cysteine (%) | 0.76 |

^1^Provided per kilogram of diet: vitamin A, 8.4 KIU; vitamin D, 3.0 KIU; vitamin E, 54.90 mg; vitamin K, 2.70 mg; vitamin B_1_, 1.93 mg; vitamin B_2_, 7.92 mg; vitamin B_6_, 4.70 mg; vitamin B_12_, 0.04 mg; niacin, 50.30 mg; folic acid, 1.30 mg; pantothenic acid, 15.73; biotin, 0.20 mg; manganese, 83.20 mg; zinc, 93.60 mg; iron, 122.4 mg; iodine, 0.40 mg; copper, 10.00 mg; selenium, 0.39 mg; cobalt, 0.15 mg.

**Table S2** Forward and reverse primer sequences for PCR analysis

| Gene | Accession number | Primer sequences, 5′ to 3′ | Product size, bp |
| --- | --- | --- | --- |
| β-actin | L08165 | F: ATTGTCCACCGCAAATGCTTC | 113 |
|  |  | R: AAATAAAGCCATGCCAATCTCGTC |  |
| CYP7A1 | NM_001001753 | F: AGGAGGCAATGAGGCTATCG | 229 |
|  |  | R: CCGTTGCGGTAGAAGTCAGT |  |
| CYP8B1 | NM_001389480 | F: CATCATTCCCTGGCTGGGTT | 225 |
|  |  | R: TAGCCAAAAACCCGGAGGAC |  |
| CYP27A1 | XM_040676620 | F: GCTCAGGACTTTCGTCTGGC | 112 |
|  |  | R: GTGGCCAAAGGTTGACTTCC |  |
| CYP7B1 | XM_040664211.2 | F: CGGCTGACTGGGAACAACTT | 142 |
|  |  | R: GCACTTTGTGGCCATCTTGG |  |
| BAAT | XM_004941276.5 | F: TGGACAACTCGGCCATCTTC | 95 |
|  |  | R: CACGAAGAGGACCTTGCCTT |  |
| BSEP | XM_040676673.2 | F: TGTTGGTGCTAGTGGAGCTG | 282 |
|  |  | R: TGCTGTGGCAAGTCCATGAT |  |
| NTCP | XM_040671950 | F: GCATAATGCCCTTGACAGCA | 216 |
|  |  | R: TTCCCACATTGGCCACAGAC |  |
| FXR | NM_001030855.2 | F: GAGCGTGAGGAAGAACCACA | 89 |
|  |  | R: TGCAGTATCGGCACTGGTTT |  |
| VDR | XM_046934193.1 | F: GAAAGCTGTTTCGTGGTGCT | 187 |
|  |  | R: CGGACAGGTGAACATCGCTT |  |
| SHP | NM_001030893.3 | F: ACGCACTGAGCTACAGACAC | 113 |
|  |  | R: AGGGAGCTTTCCAGACATGC |  |
| LRH-1 | NM_205078.2 | F: CCGCCTGCAAACCGAC | 196 |
|  |  | R: CAGCACAGGTTTAGCGGGA |  |
| FGF19 | NM204674 | F: TTCGTCCAGACGGCTACAAC | 149 |
|  |  | R: CTCCACTGGCACAGTGTTGA |  |
| FGFR4 | XM_040682923.2 | F: GGAGATGGAGCCAGACTCG | 228 |
|  |  | R: TTCTTGAACCAGCGGATGCT |  |
| ASBT | NM_001319027 | F: TGATGATCATGGGATGCTGT | 202 |
|  |  | R: ATTCCAATGCTGTCGTAGGG |  |
| OSTα | NM_001277697.1 | F: GGCAGATGATCCCAGGTTCC | 218 |
|  |  | R: AGCTGGTCTTGCGGTAGATG |  |
| SREBP-1c | XM_046927256 | F: GCCCTCTGTGCCTTTGTCTTC | 130 |
|  |  | R: ACTCAGCCATGATGCTTCTTC |  |
| C/EBPα | NM_001031459 | F: GACATCTGCGAGAACGAGCA | 154 |
|  |  | R: GCATGCCGTGGAAATCGAAA |  |
| FABP4 | NM_204290 | F: GCCTGACAAAATGTGCGACC | 130 |
|  |  | R: ATTAGGCTTGGCCACACCAG |  |
| ELOVL6 | XM_046916529 | F: GGTGGTCGGCACCTAATGAA | 169 |
|  |  | R: TCTGGTCACACACTGACTGC |  |
| SCD1 | NM_204890 | F: GTTTCCACAACTACCACCATACATT | 175 |
|  |  | R: CCATCTCCAGTCCGCATTTT |  |
| ATGL | NM_001113291 | F: TCCTAGGGGCCTACCACATC | 195 |
|  |  | R: CCAGGAACCTCTTTCGTGCT |  |
| CPT-1 | XM_046918285 | F: TAGAGGGCGTGGACCAATAA | 229 |
|  |  | R: TGGGATGCGGGAGGTATT |  |
| LPL | NM_205282 | F: CCGATCCCGAAGCTGAGATG | 186 |
|  |  | R: ACATTCCTGTCACCGTCCAC |  |
